# Improvement in Mechanical Properties of Al2024 Alloy Using Mechanical Working and Heat Treatment

**DOI:** 10.3390/ma16165568

**Published:** 2023-08-10

**Authors:** Zhengfeng Zhu, Renbao Qin, Yishan Sun, Jie Tang, Fulin Jiang, Chuang You

**Affiliations:** 1 CRRC Qishuyan Institute Co. Ltd., Changzhou 231011, China; zhuzf9732@163.com (Z.Z.); youchuang.qs@crrcgc.cc (C.Y.); 2 College of Materials Science and Engineering, Hunan University, Changsha 410082, China; 13548657660@163.com (R.Q.); tangj94@hnu.edu.cn (J.T.)

**Keywords:** aluminum alloy, repetitive continuous extrusion forming, heat treatment, mechanical properties, microstructure

## Abstract

Extrusion speed has a significant influence on the extrusion temperature, microstructure and mechanical properties of the material in the repetitive continuous extrusion forming (RCEF) process. In this work, the mechanical properties of Al2024 were improved by adjusting the speed (with a general range of 2–10 rpm) of repetitive continuous extrusion and applying subsequent heat treatment. During the RCEF process, an increase in the extrusion speed from 4 to 8 rpm was found to increase the extrusion temperature and then enhance the solid solution function. The grain size was affected by the combined effect of deformation speed and its induced temperature. A high-strength Al2024 (ultimate tensile strength of 497.6 MPa) with good elongation (12.93%) was obtained by increasing the extrusion speed and conducting solid solution and artificial aging treatments. The main strengthening mechanisms could be attributed to finer grain size and a larger amount of *S* (Al_2_CuMg) precipitates.

## 1. Introduction

The Al2024 is a typical Al-Cu-Mg-series light-weight structural material and has extensive applications in aerospace, automobile manufacturing, and other fields due to its low density, high specific strength, high specific modulus, good processing performance, heat-treatability, etc. [1,2,3,4,5,6,7,8]. With the development of modern industry, higher requirements for the comprehensive mechanical properties of Al alloys have been proposed. Under this background, many manufacturing methods have been designed and employed to produce high-performance Al alloys. Severe plastic deformation (SPD) is one of the most promising processes as it can refine the grain size to the micron and even nanometer scale [9,10,11,12]. SPD technology was first introduced by PW Bridgeman through a combination of high hydrostatic pressure and shear deformation [13]. Subsequently, a large number of SPD methods were devised and applied to promote grain refinement and enhance strength. The representative techniques are equal-channel angular processing (ECAP) [14], high-pressure torsion (HPT) [15,16], and repetitive continuous extrusion (RCEF) [17,18]. In this article, RCEF technology was applied to produce a high-performance Al2024 as this technology can withstand great strain, enabling us to refine the grain size to improve mechanical properties.

The RCEF process has been used to produce other Al alloys in recent years. For example, Kong et al. [18] improved the continuous extrusion die and employed the RCEF process for multi-pass deformation of an Al-Fe-Cu alloy, revealing that dynamic recrystallization occurred with an increase in the number of extrusion passes. This phenomenon led to a significantly refined grain size and improved performance. Hu et al. [19] investigated the microstructure and mechanical properties of an Al-Mg-Si alloy during the RCEF process and subsequent heat treatment. The tensile strength and elongation of the alloy were significantly enhanced after the seventh pass of RCEF. Apart from grain refinement, RCEF also has other advantages such as introducing many defects such as dislocations and vacancies, which can help improve strength. However, these advantages are weakened after solid solution (SS) treatment due to the disappearance of grain growth and corresponding defects. In addition, the quenching operation for large coil heavy alloys is difficult and requires expensive equipment. In addition, the microstructures and mechanical properties of the center and outer portions of large coils are uneven due to the uneven heating and cooling rate. Therefore, in this study, we analyze short-flow processing technology without additional SS treatments. To achieve this objective, the most crucial approach is to introduce a high level of supersaturated solid solubility before SS treatment. Specifically, two main methods are used by researchers: (i) improving initial supersaturated solid solubility via pre-heating treatment and (ii) improving online solid solubility by increasing the extrusion temperature [20]. For the Conform process, we seek to improve the online solid solubility by increasing extrusion speed to increase the extrusion temperature. Zhao et al. [21] conducted experiments and numerical simulations to analyze the continuous extrusion of AA6063 Al alloy under different extrusion speeds, finding that the extrusion temperature mainly depends on deformation speed, which determines the mechanical properties. It was also reported [22] that the increase in temperature caused by increased extrusion speed can accelerate the formation of metallurgical bonding and thus improve the strength and elongation of extruded AA 6063 Al alloy. Therefore, it is feasible to achieve higher online solubility by increasing the extrusion speed. In this work, the influence of extrusion speed on the microstructure and mechanical properties of Al2024 during the RCEF process was investigated. The functions of different heat treatments on the mechanical properties of the processed material were also examined, which could provide important guidance for developing short-flow processing technology.

## 2. Materials and Methods

The Al2024 used in this study was obtained using a continuous casting process with a main chemical composition of 4.4 Cu, 1.5 Mg, 0.6 Mn, 0.1 Cr, 0.25 Zn, 0.5 Si, 0.12 Ti, 0.1 Fe (wt.%). The as-cast rods with a diameter of 16 mm were immediately water-cooled at a cooling rate of around 100 °C/s after being drawn out of the crystallizer. The ultimate tensile strength and elongation of cast alloy were 311.2 MPa and 19.1%, respectively. The as-cast rods were then subjected to repetitive continuous extrusion forming (RCEF) with a total of 3 passes using an LJ300 continuous extrusion machine. During the RCEF process, two different extrusion wheel speeds (i.e., 4 and 8 rpm) were adopted to explore the effect of extrusion speed on the microstructural characteristics and mechanical properties of the Al2024. The deformation conditions were identical and yielded no change in size or shape during the 3 processing passes. The extruded alloys were water quenched immediately after each Conform pass and then subjected to different heat treatments, including direct artificial aging (T5 treatment) at 180 °C and artificial aging at 180 °C after solid solution treatment at 500 °C for 2 h, followed by water quenching (T6 treatment). The detailed abbreviation of the specimens and the corresponding parameters are summarized in Table 1.

Selected specimens were cut from the center portion of the extruded rods along the extrusion direction (ED) to examine the microstructural characteristics and mechanical properties. Vickers microhardness measurements were performed using an HVS-1000 sclerometer with a load of 0.2 kgf and a dwell time of 15 s. To ensure the accuracy of the hardness measurement, 10 points in different regions were tested for each sample to obtain the mean value and standard deviation. Specimens for tensile tests were machined according to the ASTM-E8 and then subjected to a computer-controlled INSTRON3382 universal testing machine with a strain rate of 2 × 10^−3^ s^−1^ for uniaxial tensile tests. For each tensile test, 3 samples were prepared and tested to guarantee the reliability of results. The samples were mechanically polished before being etched with Keller’s reagent and observed using CK-300 optical microscopy (OM). The polished specimens were also observed with a TESCAN MIRA field emission scanning electron microscope (FSEM). For EBSD analysis, the selected samples were ground and electropolished in an electrolyte consisting of 20% HClO4 and 80% C2H5OH under −35 °C for 18 s to remove the damage caused by grinding and mechanical polishing. Subsequently, EBSD measurements were performed on a JEOL-7900FSEM system equipped with an Oxford Instruments Nordlys Nano EBSD detector. The EBSD data were analyzed using orientation imaging microscopy (OIM) software. After mechanical thinning and double-jet electro-polishing in a 30% nitric acid and 70% methanol solution at −20 °C, the samples for the TEM test were transformed into foils with a thickness of 80 μm and a diameter of 3 mm. The TEM observations were performed using an FEI Tecnai G2 F20 TEM system.

Three-dimensional elastoplastic finite element analysis (FEA) based on ABAQUS software was carried out to simulate the Conform process. The geometric dimensions of the die, groove roll, and workpiece were identical to the experimental parameters to assure the results were reliable. The material properties of FEA were summarized in Table 2. The initial die temperature was assumed to be ~150 °C. The heat transfer coefficient between the die and workpiece was 30 N/sec·mm· °C. The Young’s modulus and Poisson’s ratio of the workpiece were 70,000 MPa and 0.33, respectively. The friction coefficient between the workpiece and groove wheel was defined as 0.95, and the lubricated friction coefficient on the die surface was set to 0.4 [21,23]. A penalty function was used. The extrusion die was considered a rigid body, while the workpiece was a rigid-plastic body meshed into 400,000 three-node grids which were located in the stable range and had little on the final simulated results.

## 3. Results and Discussion

### 3.1. Finite Element Analysis of Continuous Extrusion Formation

**Figure 1:**
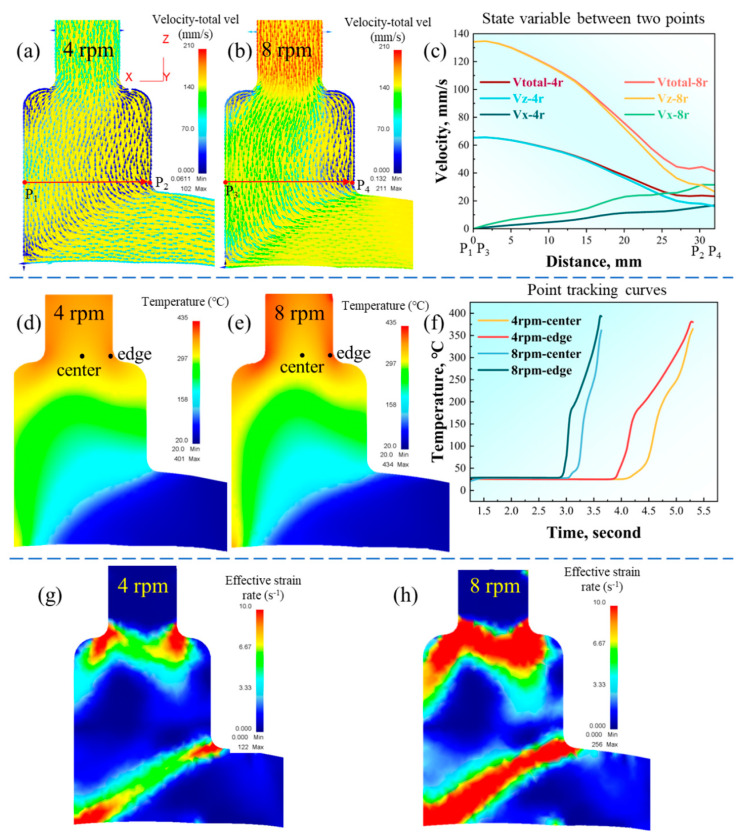
Finite element simulation results of the Conform process: (**a**–**c**) flow velocity distributions; (**d**–**f**) deformation temperature distributions; (**g**,**h**) effective strain rate distribution.

Figure 1 shows the finite element simulation results for the continuous cast Al2024 during the Conform process with different extrusion speeds. The metal flow behavior simulations are illustrated in Figure 1a–c, where the direction and shade of the arrows represent the metal flow direction and speed, respectively. The metal has a flow velocity in both the X and Z directions. After the mold cavity was filled, the flow direction changed from horizontal to vertical, and the flow velocity slowed. The velocity distribution in the mold was severely uneven because of friction. The velocity on the right side near the cavity wall was very slow, which is the dead zone for metal flow during Conform. Driven by the rotation of the extrusion wheel, the velocity near the extrusion wheel (P1 and P3) was much higher than that on the other side (P2 and P4). As a result, there was an obvious velocity gradient distribution between these two sides, which can be seen in the variable distribution model in Figure 1c. Comparing the velocity fields under different extrusion wheel speeds shows that the higher the rotation speed of the extrusion wheel, the faster the metal flow velocity. Under these conditions, the uniformity of the velocity distribution will also increase. Here, the velocity difference between P1 and P2 is about 64.4%, while that between P3 and P4 is slightly higher (66.7%).

The temperature field distribution obtained using the finite element simulation of Conform is shown in Figure 1d,e. In this simulation, the initial billet was at room temperature before entering the extrusion process. Friction between the billet and the extrusion wheel groove and plastic deformation in the deformation cavity gradually increased the temperature of the continuous extrusion system. The temperature increased rapidly, especially after undergoing severe shear deformation. After a certain period, the whole system reached the stage of thermal balance, that is, the stage of stable continuous extrusion. At this stage, the temperature of the billet, extrusion wheel, and die in the system remained largely stable. The temperature achieved its maximum value when it reached the die outlet, as shown in Figure 1d,e, where the maximum value at 4 rpm is ~401 °C, while that at 8 rpm is ~434 °C. An increase in extrusion speed will increase the thermal effect and thus the temperature of the continuous extrusion system. Notably, a higher outlet temperature will enhance the solid solution effect, and the high temperature induced by increasing the extrusion speed can accelerate the formation of metallurgical bonding. The point tracking curves in Figure 1f illustrate the temperature variation of the center and edge regions under two extrusion speeds, from the billet at room temperature to the exit of the mold. In Figure 1f, the temperature at the center is lower than that at the edge because the edge is subjected to greater friction and generates more heat.

Figure 1g,h show the effective strain rate distributions obtained via finite element simulation. Here, the effective strain rate increases when the rotational speed increases from 4 to 8 rpm, with maximum values of 122 and 256 s^−1^, respectively. The increase in the equivalent strain rate is also helpful for metallurgical bonding. The deformation condition of Conform, however, is relatively special and uneven. The strain rates between the center and edge portions are also uneven. Severe shear deformation bands (IISBs) are found under extrusion speeds of both 4 rpm and 8 rpm. The existence of IISB can improve non-uniform deformation and generate significant amounts of heat, thereby achieving hot deformation without an external heat source and improving the plastic’s working performance. In addition, the IISB observed under 8 rpm was much more obvious than that under 4 rpm.

### 3.2. Microstructural Characteristics of the Initial Continuous Cast Al2024

**Figure 2:**
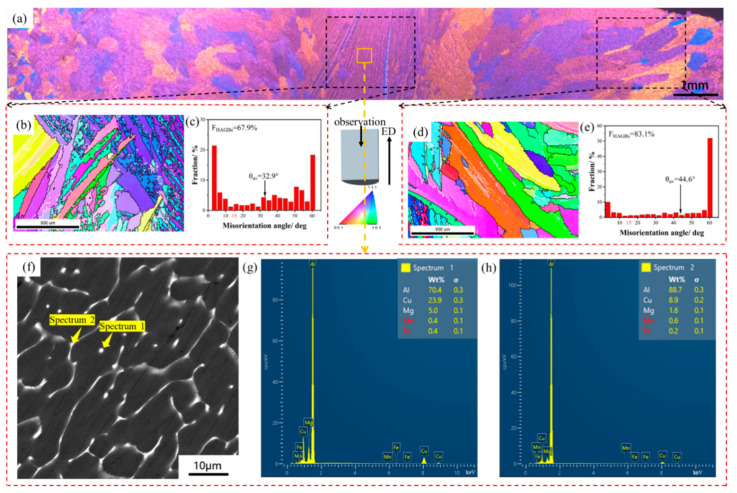
Initial microstructure of the continuous cast Al2024: (**a**) OM image; (**b**,**d**) EBSD inverse pole figures (IPFs); (**c**,**e**) misorientation angle distribution images; (**f**) SEM graph and (**g**,**h**) EDS results of Spectra 1 and 2.

The initial microstructure of the continuous cast Al2024 is shown in Figure 2. In Figure 2a, the optical microstructure crosses the entire section and shows inhomogeneous grains formed during continuous casting. Much finer grains are indicated in both center and edge areas. In the EBSD inverse pole figures (IPFs), shown in Figure 2b,d, the white lines are used to represent low angle grain boundaries (LAGBs, 2~15°), whereas the black lines correspond to high-angle grain boundaries (HAGBs, >15°). As shown in Figure 2a,b,d, the grains are elongated as a result of the fast cooling rate during continuous casting and the drag force of the traction device. The microstructure is uneven between the center and edge regions along the diameter. As shown in Figure 2b,c, the center region is full of dendrite structures with a 67.9% proportion of HAGBs (FHAGBs) and an average misorientation angle (*θ*_av_) of 32.9°. The grains in the edge portion are also elongated but much coarser than those in the center portion. As shown in Figure 2d,e, the FHAGBs and *θ*_av_ are 83.1% and 44.6°, respectively. The second phase is segregated along the grain/subgrain boundaries before joining in a network in the SEM image (Figure 2f). According to the Energy Dispersive Spectroscopy (EDS) results in Figure 2g,h, the main elements segregated along the boundaries are copper (Cu) accompanied by a small amount of magnesium (Mg). This phenomenon is common as Cu and Mg are easy to enrich and form eutectic phases due to their low melting points. The main composition of the eutectic phases was determined as α (Al) + *S* (Al_2_CuMg).

### 3.3. Microstructural Evolution during the RCEF Process

**Figure 3:**
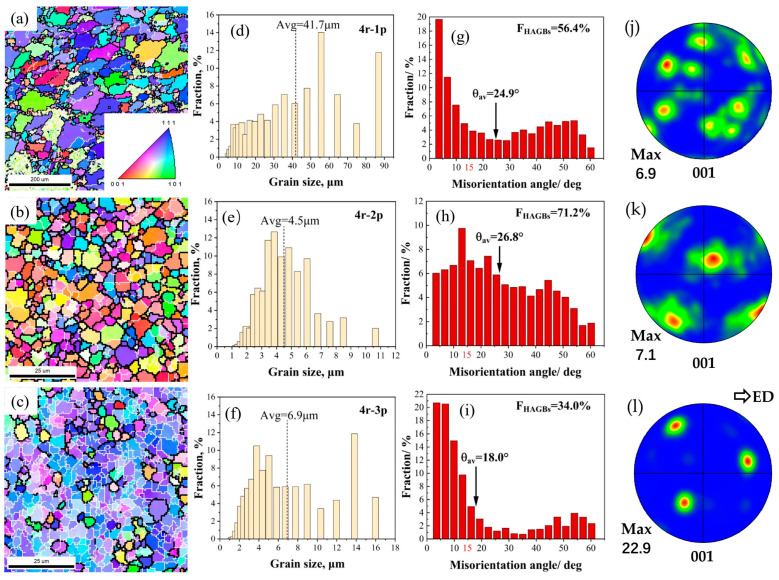
EBSD maps of RCEF samples at an extrusion speed of 4 rpm with different passes (i.e., 1p, 2p, 3p): (**a**–**c**) IPF micrographs; (**d**–**f**) statistical maps of grain size; (**g**–**i**) misorientation angle distribution of grain boundaries; and (**j**–**l**) pole figures showing the texture.

**Figure 4:**
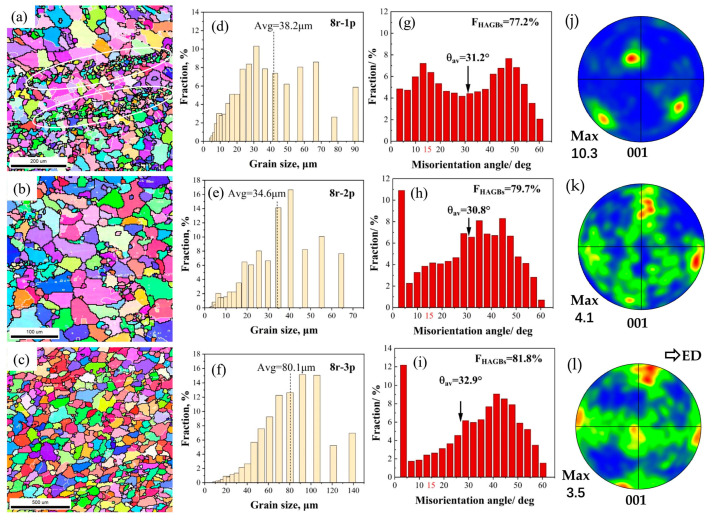
EBSD maps of RCEF samples at an extrusion speed of 8 rpm with different passes (i.e., 1p, 2p, 3p): (**a**–**c**) IPF micrographs; (**d**–**f**) statistical maps of grain size; (**g**–**i**) misorientation angle distribution of grain boundaries; and (**j**–**l**) pole figures showing the texture.

Figure 3 and Figure 4 present the EBSD microstructures of Al2024s under different extrusion speeds (i.e., 4 rpm and 8 rpm) processed by different RCEF passes, where the grain size was summarized in Table 3. For the sample deformed at 4 rpm during the first pass (4r-1p), a duplex microstructure can be observed in Figure 3a. Some coarse grains containing abundant substructures are distributed in the matrix, while some much finer recrystallized grains surrounded by HAGBs are non-uniformly located around the coarse ones. As shown by the static grain size (Figure 3d), the average grain size (Avg) of the 4r-1p sample was ~41.7 μm, which represents a significant refinement of over 10 times compared to the as-cast alloy. In the misorientation distribution of grain boundaries (Figure 3g, LAGBs in the 4r-1p sample are more numerous than those in the as-cast alloy, with FHAGB and *θ*_av_ values of only 56.4% and 24.9°, respectively. Here, as the RCEF pass increases, the grains experience a larger strain and plastic deformation, eventually fragmenting into finer grains. As shown in Figure 3b, the grains of the sample deformed at 4 rpm during the second pass (4r-2p) were refined by nearly 9.3 times compared to the 4r-1p sample, with a value of ~4.5 μm. The FHAGBs and *θ*_av_ also increased to 71.2% and 26.8°, respectively. The grain size of the sample deformed at 4 rpm after the third pass (4r-3p) and slightly increased to ~6.9 μm, while the FHAGBs and *θ*_av_ decreased to 34.0% and 18.0°, respectively. Many LAGBs were observed, especially those at 3.675° and 6.725°, which totaled more than 40%. A similar phenomenon was reported by Hu et al. [19] for the RCEF process of the Al-Mg-Si alloy, wherein the FHAGBs decreased largely because of the high content of LAGBs at 5.575° after the fourth and seventh extrusion passes. In addition, the pole figures (Figure 3j–l) show the gradually enhanced texture along the extrusion direction.

For the specimens deformed at 8 rpm during the first pass (8r-1p), a mixed grain configuration was observed, as shown in Figure 4a. The white circle shows some fine clustered grains, which may have resulted from the breakdown of larger grains. Corresponding to the mixed microstructure, the statistical graph of grain size (Figure 4d) shows typical bimodal characteristics, and the Avg (~38.2 μm) is close to that of the 4r-1p sample. The FHAGBs and *θ*_av_ (Figure 4g) of the 8r-1p sample were higher than those of the 4r-1p sample (i.e., 77.2% and 31.2°. respectively), which indicates an improvement in DRX. The mixed grain microstructure was retained in the sample deformed at 8 rpm during the third pass (8r-3p) with a slightly lower Avg of ~34.6 μm, but the portion of finer grains decreased significantly, as shown in Figure 4e,h. The FHAGBs and *θ*_av_ were relatively close to those of the 8r-1p sample, with 79.7% and 30.8°, respectively. After being deformed at 8 rpm during the third pass (8r-3p), the grain morphology became more uniform and equiaxed, but the grain size increased to ~80.1 μm (Figure 4c,f). The FHAGBs and *θ*_av_ increased to 81.8% and 32.9°, respectively. This grain morphology and higher FHAGBs indicate the advancement of DRX compared to prior passes. Comparing Figure 4j–l with Figure 3j–l, the texture is clearly weakened with increased extrusion speed.

### 3.4. Mechanical Properties of RCEF Samples after Different Heat Treatments

**Figure 5:**
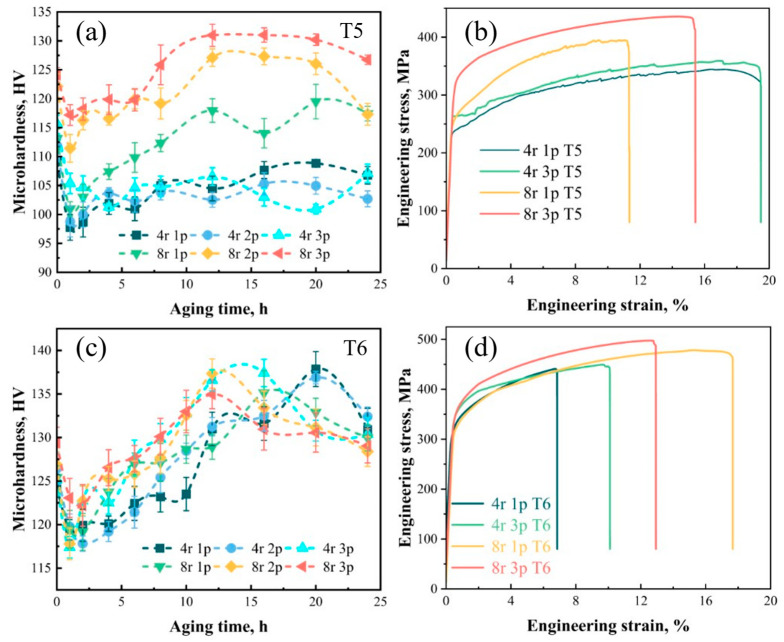
Mechanical properties of RCEF samples deformed at different speeds and passes: (**a**) Vickers hardness variation during T5 treatment; (**b**) engineering stress–engineering strain curves of selected T5-treated samples; (**c**) Vickers hardness variation during T6 treatment; (**d**) engineering stress–engineering strain curves of selected T6-treated samples.

T5 and T6 treatments were carried out on the RCEF alloys to explore the effects of aging treatment on the mechanical properties. The Vickers hardness variation of alloys deformed at different extrusion speeds and passes during T5 treatment is summarized in Figure 5a. For the samples after extrusion (i.e., an aging time of 0 h), the hardness increased from 109.1 to 115.6 HV as the extrusion pass increased from 4r-1p to 4r-3p and from 113.1 to 124.1 HV as the pass increased from 8r-1p to 8r-3p. The hardness after extrusion was positively affected by the extrusion pass and speed. For the samples deformed at 4 rpm, there was little difference in peak hardness between the three passes, with values of 108.8, 105.3, and 107.1 HV, respectively. Interestingly, the peak hardness of the samples deformed at 4 rpm was lower than the extruded state. This result is related to the low supersaturation solubility caused by the low outlet temperature after extrusion. However, the outlet temperature of alloys deformed at 8 rpm was higher, thus causing a greater online solution effect and enhancing the aging response. The peak hardness of aging was positively correlated with the number of passes, with the peak hardness increasing from 119.5 to 131.0 HV as the pass increased. Tensile tests were performed on samples aged for 16 h in order to unify the variation of aging time, as shown in Figure 5b and Table 4. The ultimate tensile strength (UTS) and elongation of the 4r-1p-T5 sample were 344.5 MPa and 19.50%, respectively. The 4r-3p-T5 sample deformed at the same speed but during the third pass presented a higher UTS (357.4 MPa) and equivalent elongation (19.48%). Corresponding to the hardness results, samples deformed at 8 rpm achieved a higher UTS after T5 treatment, with values of 391.9 (8r-1p-T5) and 434.6 MPa (8r-3p-T5), whereas elongation was lower than that of samples deformed at 4 rpm, with 11.43% and 15.43%, respectively. T6 treatment more significantly increased strength for all specimens, as shown in Figure 5c,d. The peak hardness of T6-treated specimens deformed at different speeds and passes was higher than that of T5-treated samples. The values were within a relatively small range from 135.0 to 137.9 HV. Unified heat treatment (i.e., 500 °C/2 h + 180 °C/16 h) was adopted for all samples before being subjected to tensile tests, and the test results are shown in Figure 5d and Table 4. The general rule of tensile strength is like that of the T5 state, which significantly increases with increases in extrusion speed and passes. The UTS of 4r-1p and 4r-3p samples were 441.0 and 445.8 MPa with low elongation of 6.85% and 10.10%, respectively. However, the tensile strength of the 8r-1p and 8r-3p samples was much higher than that of samples extruded at 4 rpm, reaching 465.7 and 497.6 MPa, respectively. In addition to the high strength of these samples, their elongation results were also superior, with values of 17.68% and 12.93%, respectively. Therefore, an increase in the extrusion speed and passes enhanced the mechanical properties of RCEF specimens, and T6 treatment (500 °C/2 h + 180 °C/16 h) was able to improve not only strength but also elongation.

### 3.5. Microstructures of RCEF Samples after Different Heat Treatments

#### 3.5.1. SEM and EBSD Microstructures of RCEF Samples after AA Treatment

**Figure 6:**
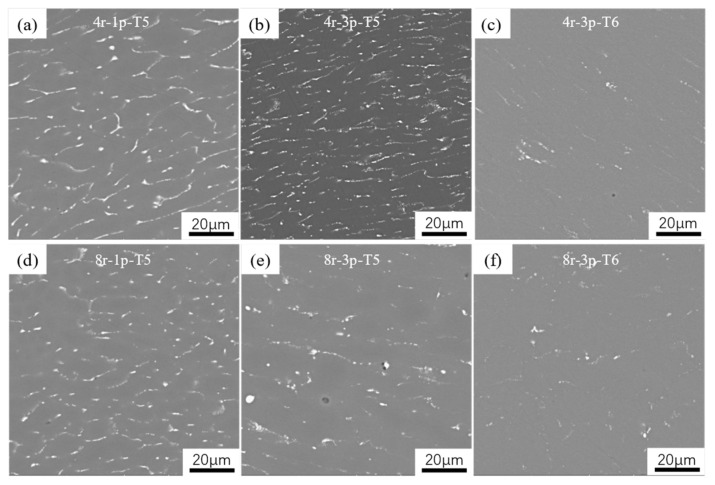
SEM microstructures of selected samples after AA treatment: (**a**) 4r-1p-T5; (**b**) 4r-3p-T5; (**c**) 4r-3p-T6; (**d**) 8r-1p-T5; (**e**) 8r-3p-T5; (**f**) 8r-3p-T6.

SEM images of aged Al2024s with different extrusion speeds and passes are shown in Figure 6, which illustrates the morphology and distribution of the secondary phases. The aging time for the selected samples was 16 h, as per the hardness curve. Notably, the aging treatment only affected the presence and distribution of small precipitates in the samples, rather than the coarse second phases. Comparing the T5-treated samples with the same pass but using different speeds (Figure 6a vs. Figure 6d; Figure 6b vs. Figure 6e) demonstrates that the solid solution effect increased as the speed increased. On the other hand, samples extruded at the same speed but with different passes (Figure 6a vs. Figure 6b; Figure 6d vs. Figure 6e) present increased solid solution effects with an increase in the past. With an increase in extrusion speeds and passes, the coarser dendritic second phases in the continuous cast alloy gradually break and refine as a result of frictional heat effect and strain action, resulting in a better online solution effect able to better enhance the mechanical properties after AA treatment. Thus, the differences in online solution effects may be the main reason for the differences in the mechanical properties of these T5-treated specimens. On the other hand, the second phase in the samples was largely dissolved into the matrix after T6 treatment, indicating that the effect of SS treatment was very significant. This result is another important reason for the significant increase in the performance of T6-treated samples compared to that of the same alloy after T5 treatment.

**Figure 7:**
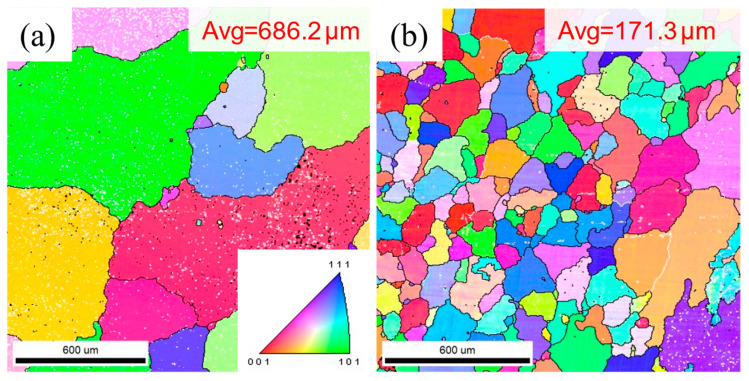
EBSD IPF maps of selected samples after SS treatment: (**a**) 4r-3p; (**b**) 8r-3p.

EBSD IPF maps of selected samples (i.e., 4r-3p and 8r-3p) after SS treatment are presented in Figure 7. The grains of the 4r-3p sample after SS treatment (Figure 7a) wasfound to be extremely coarse (~686.2 μm), whereas the grain size of the 8r-3p sample after SS treatment (Figure 7b) was much finer (~171.3 μm). As AA treatment after the SS treatment did not change the grain size, it is reasonable to infer that the 8r-3p-T6 sample has better comprehensive mechanical properties from the perspective of fine grain strength.

#### 3.5.2. TEM Images of RCEF Samples after AA Treatment

**Figure 8:**
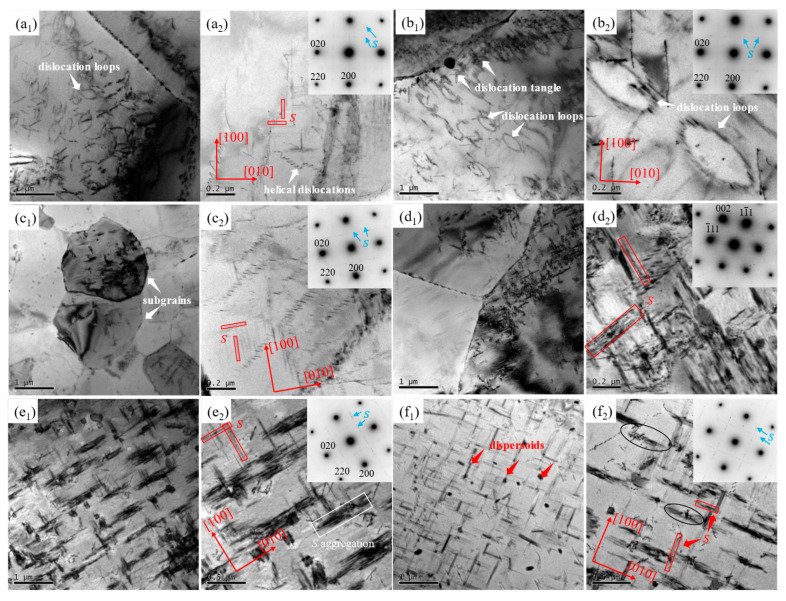
TEM microstructures of selected samples after AA treatment: (**a1**) and (**a2**) 4r-1p-T5; (**b1**) and (**b2**) 8r-1p-T5; (**c1**) and (**c2**) 4r-3p-T5; (**d1**) and (**d2**) 8r-3p-T5; (**e1**) and (**e2**) 4r-3p-T6; (**f1**) and (**f2**) 8r-3p-T6.

To better understand the reasons underlying the alloy strengthening and toughening mechanism, TEM was performed, as shown in Figure 8. Many dislocation loops were observed in the 4r-1p-T5 (Figure 8(a1)) and 8r-1p-T5 (Figure 8(b1)) samples. Other dislocation morphology was also observed in Figure 8(a2,b2), such as helical dislocations. In addition, the main strengthening phase (i.e., the *S* phase) and its diffraction spots were observed to occur in Figure 8(a1). The *S* phases exist in the form of thicker needle shapes perpendicularly distributed along the <100> and <010> directions with light contrast. Although the precipitate size of the 4r-1p-T5 sample (~102.4 nm) was the finest among all the samples, its density was very low and thus did not contribute significantly to the aging-based hardening. For samples deformed after the third pass (Figure 8(c1,d2)), subgrains formed along with *S* precipitates. *S* precipitates in 4r-3p-T5 (Figure 8(c2)) were also distributed along the <100> and <010> directions with an average size of ~185.0 nm. The low density of precipitates did not notably improve mechanical performance. However, the density of *S* precipitates in the 8r-3p-T5 sample (Figure 8(d2)) was obviously higher with an average size of ~308.1 nm. Although the precipitate size was coarser, its high density may be the main reason that the 8r-3p-T5 sample had the highest strength among all T5-treated samples. The microstructure of the 4r-3p-T6 sample is shown in Figure 8(e1,e2)). There were no obvious grain/subgrain boundaries in samples after the T6 treatment, indicating that the grain size may be coarse. The *S* phases in Figure 8(e2)) are still oriented along the <100> and <010> directions with an average size of ~396.9 nm. However, many *S* phases were found to aggregate together (white box) and become non-uniformly distributed. For the 8r-3p-T5 sample (Figure 8(f1,f2)), the distribution of *S* precipitates was more uniform, and the average size was ~573.9 nm. A coarse precipitate size did not benefit the material’s mechanical properties; thus, the uniformity of the *S* phases may be the real explanation for not only the superior strength but also the greater elongation of the material. In addition, many dispersed secondary particles were found in the matrix and presented a rod-like or short rod-like morphology. These dispersoids were found to interact with *S* precipitates and dislocations, as indicated by circles in Figure 8(f2). These particles may be retained during SS treatment as there were few particles in the sample without SS treatment (i.e., 8r-3p-T5).

### 3.6. Discussion

The results indicated that extrusion speed and pass had an obvious impact on the deformation behaviors, microstructures, and mechanical properties in this study. However, the deformation speed itself did not impact the grain size, which was determined by the stored energy of deformation. The stored energy of deformation was mainly influenced by the deformation rate, strain accumulation, and temperature [24,25]. The simulation results indicated that variations in the extrusion speed directly affected deformation behaviors and significantly influenced the maximum outlet temperature of the RCEF process. On the one hand, a higher speed yielded a higher strain rate (Figure 2g,h) and thus higher stored energy. According to Sweet et al., this increase in stored energy could significantly improve the nucleation rate of small recrystallized grains [26]. On the other hand, the higher speed produced a higher outlet temperature (Figure 1d,e), resulting in increased grain size due to the acceleration of grain growth at high temperatures. In other words, deformation and temperature exerted opposing influences on the microstructure. For the RCEF process, the extrusion pass also influenced the microstructure by increasing stored energy through accumulating equivalent strain and coarsening the grain size via the extrusion temperature. When the extrusion wheel velocity was 4 rpm, the grain refinement accomplished through the accumulation of strain dominated the microstructure’s evolution, while the grain coarsening caused by the extrusion temperature was weak because of the relatively low extrusion temperature. As the extrusion pass increased to the second pass, the grain size was greatly refined by 89% from 41.7 to 4.5 μm (Figure 3d,e). After the third pass, the grain size slightly increased to 6.9 μm, which indicated the limited role of a low outlet temperature in this process (Figure 3f). In contrast, a high outlet temperature played a key role in grain size evolution when deformed at 8 rpm. The grain size of the 8r-1p sample (Figure 4d, 38.2 μm) was 9% lower than that of the 4r-1p sample as a result of the higher effective strain rate. However, the grain sizes of the 8r-2p and 8r-3p samples were 34.6 and 80.1 μm (Figure 4e,f), 7.7 and 11.6 times greater than those of the 4r-2p and 4r-3p samples, respectively. The coarse microstructures indicated that the high extrusion temperature dominated the resulting grain size at higher extrusion speeds and resulted in an obvious increase in grain size.

The mechanical properties of samples directly subjected to AA treatment (i.e., T5 treatment) were influenced by the deformation speed and pass. However, generally, the deformation speed and pass did not, themselves, exert a dominant effect and could simply be regarded as a tool for affecting the maximum outlet temperature during the extrusion process. The high extrusion temperature helped achieve a greater solid solution effect by dissolving the second phases into a solution. The solid solution effect was observed to increase as the deformation speed and pass increased (Figure 6). Indeed, the greater supersaturated solid solubility caused by higher deformation temperatures was conducive to the formation of nano-size precipitates during AA treatment, thus improving the strength. The 8r-3p sample (Figure 8(d2)) had the largest amount of *S* precipitates and thus the best strength. As a result, the strength of the T5-treated samples gradually increased by 26.2% from 344.5 (4r-1p) to 434.6 MPa (8r-3p) with an increase in the extrusion speed and pass (Table 1). Elongation was mainly dependent on the size and homogeneity of grains [21]. Thus, the samples deformed at 4 rpm with a finer grain size experienced greater elongation (Table 1). On the other hand, the presence of *S* precipitates in large amounts with coarser sizes in the samples deformed under 8 rpm (Figure 8) strongly explained the lower elongation. It was confirmed that an increase in extrusion speed greatly improves the strength of T5-treated alloys. For example, the UTS of the 8r-3p-T5 sample was close to that of the 4r-3p-T6 sample, while the former was only 2.5% lower than the latter. This result demonstrates that an increase in extrusion speed could yield the solution caused by SS treatment. This result opens the possibility to explore short-flow processing technology without additional SS treatment.

Although an increase in extrusion speed and pass significantly improved the strength of T5-treated samples, the maximum strength was still not ideal. Additional SS treatment before AA treatment was implemented to further increase the mechanical properties. The mechanical properties of T6-treated samples were also found to increase as the deformation speed and pass increased. Notably, the 8r-3p sample achieved not only extremely high strength (497.6 MPa) but also good elongation (12.93%) that were, respectively, 11.6% and 28.0% higher than the values of the 4r-3p sample. The main mechanisms underlying the strength and fortification of the 8r-3p-T6 sample could be assigned to three factors. First, the 8r-3p-SS sample had a much finer grain size than the 4r-3p-SS sample (Figure 7). It is well known that fine grain strengthening is the only method that can improve both strength and toughness. This principle can be described as the Hall-Petch formula [27,28]:

(1) $$\sigma_{S}=\sigma_{0}+kd^{-\frac{1}{2}}$$

where *σ*_S_ and *d* represent the yield strength and average grain size, respectively, and *σ*_0_ and *k* are the materially dependent constants. The results indicated that the strength increased as the grain size decreased. Second, the original particles from SS treatment in the 8r-3p-T6 sample (Figure 8(f1,f2)) are believed to play a key role in enhancing mechanical properties. It was previously noted [29,30,31,32,33] that the dispersoids could strengthen the alloy through the Orowan mechanism. Due to being incoherent with the Al matrix, the interface between dispersoids and the Al matrix had high energy and provided a preferential nucleation position for the *S* phases [4,8,34,35,36]. Furthermore, the particles themselves could hinder dislocation movement and pinned grain boundary migration and thus impede grain growth [24,32,37,38]. The interactions between the particles, dislocations, and *S* precipitates (Figure 8(f2)) verified the above hypothesis.

Finally, a large amount of *S* precipitates were present in the matrix of the 8r-3p-T6 sample (Figure 8(f1,f2)). As the dominant phase that strengthened precipitates in the Al2024, the *S* phase was difficult to directly precipitate as it was not coherent with the matrix. The samples were generally precipitated through the following sequence: supersaturated solid solution (SSS) → Cu-Mg cluster → GP → *S*′ → *S* [1,39,40,41,42]. It was previously noted that the formation of *S* precipitates is promoted by second-phase particles and SS treatment before AA treatment. In addition, the morphology of the precipitates in the 8r-3p-T6 sample was more uniform than that of the precipitates in the 4r-3p-T6 sample. This morphology could explain the enhanced mechanical properties observed in this study, especially the elongation.

## 4. Conclusions

In this work, the effects of extrusion speed and post-heat treatment on the microstructures and mechanical properties of an Al2024 during the RCEF process were investigated The major conclusions could be summarized as follows.

(a) The maximum outlet temperature was strongly dependent on the extrusion speed. The maximum extrusion temperature of the 8 rpm extrusion process was ~434 °C, which was 33 °C greater than that of the 4 rpm extrusion process.
(b) The grain morphology was affected by the combined effect of deformation speed and its induced temperature. When the extrusion wheel velocity was 4 rpm, the grain refinement caused by the accumulation of strain dominated the microstructure’s evolution. The enhanced grain growth was observed when deformed at 8 rpm.
(c) An increase in extrusion speed greatly improved the strength of the T5-treated alloy with a maximum improvement of 24% and could compensate for the solution extent caused by SS treatment, making it possible to explore short-flow processing technology without additional SS treatment.
(d) The 8r-3p-T6 sample achieved ultra-high strength (UTS of 497.6 MPa) with good elongation (12.93%), yielding values 11.6% and 28.0% higher than those of the 4r-3p-T6 sample. The main strengthening mechanisms could be attributed to finer grain size and a larger amount of *S* precipitates.

## Figures and Tables

**Table 1:** The abbreviation of the specimens and the corresponding parameters.

Abbreviation	Extrusion Wheel Speeds, Rpm	Extrusion Pass	Heat Treatment
4r-1p-T5	4	1	Direct aging at 180 °C/16 h
4r-2p-T5	4	2	Direct aging at 180 °C/16 h
4r-3p-T5	4	3	Direct aging at 180 °C/16 h
8r-1p-T5	8	1	Direct aging at 180 °C/16 h
8r-2p-T5	8	2	Direct aging at 180 °C/16 h
8r-3p-T5	8	3	Direct aging at 180 °C/16 h
4r-1p-T6	4	1	500 °C/2 h + 180 °C/16 h
4r-2p-T6	4	2	500 °C/2 h + 180 °C/16 h
4r-3p-T6	4	3	500 °C/2 h + 180 °C/16 h
8r-1p-T6	8	1	500 °C/2 h + 180 °C/16 h
8r-2p-T6	8	2	500 °C/2 h + 180 °C/16 h
8r-3p-T6	8	3	500 °C/2 h + 180 °C/16 h

**Table 2:** Material properties for FEA.

Material Properties	Workpiece
Density, kg/m^3^	2690
Thermal conductivity, W/(m.K)	205
Specific heat, J/(kg.K)	895
Heat transfer coefficient (N/s·mm·°C)	30
Young’s modulus (MPa)	70,000
Poisson’s ratio	0.33
Penalty function	Friction factor: 0.95 (Conform wheel), 0.4 (others);
Initial billet temperature, °C	25
Initial die temperature, °C	150

**Table 3:** The summarized grain size and hardness evolutions.

Sample	Grain Size	Hardness (T5)	Hardness (T6)
4r-1p	41.7	107.7	131.6
4r-2p	4.5	105.3	132.5
4r-3p	6.9	103.0	137.4
8r-1p	38.2	114.0	133.1
8r-2p	34.6	127.3	133.4
8r-3p	80.1	131.0	135.0

**Table 4:** Tensile properties of selected samples.

Sample	YS/MPa	UTS/MPa	Elongation/%
4r-1p-T5	238.1	344.5	19.5
8r-1p-T5	256.0	391.9	11.4
Increasement fraction (4r to 8r)	7.5%	13.8%	−41.6%
4r-3p-T5	263.2	357.4	19.5
8r-3p-T5	326.4	434.6	15.4
Increasement fraction (4r to 8r)	24%	21.6%	21.1%
4r-1p-T6	325.4	441.0	6.9
8r-1p-T6	323.4	465.7	17.7
Increasement fraction (4r to 8r)	−0.6%	5.6%	157%
4r-3p-T6	349.9	445.8	10.1
8r-3p-T6	354.2	497.6	12.9
Increasement fraction (4r to 8r)	1.2%	11.6%	27.7%

## Data Availability

The data presented in this study are available on the request from the corresponding author.
